# Strategies to eliminate cervical cancer in China

**DOI:** 10.3389/fonc.2023.1105468

**Published:** 2023-06-02

**Authors:** Lu Ji, Manli Chen, Lan Yao

**Affiliations:** ^1^ School of Medicine and Health Management, Tongji Medical College of Huazhong University of Science and Technology, Wuhan, China; ^2^ School of Management, Hubei University of Chinese Medicine, Wuhan, China

**Keywords:** cervical cancer, HPV vaccination, cervical cancer screening, eliminate cervical cancer, strategies

## Abstract

Cervical cancer is a widely distributed disease that is preventable and controllable through early intervention. The World Health Organization has identified three key measures, coverage populations and coverage targets to eliminate cervical cancer. The WHO and several countries have conducted model predictions to determine the optimal strategy and timing of cervical cancer elimination. However, specific implementation strategies need to be developed in the context of local conditions. China has a relatively high disease burden of cervical cancer but a low human papillomavirus vaccination rate and cervical cancer screening population coverage. The purpose of this paper is to review interventions and prediction studies for the elimination of cervical cancer and to analyze the problems, challenges and strategies for the elimination of cervical cancer in China.

## 1 Introduction

Cervical cancer is a main threat to women’s health worldwide and contributes to widespread inequities in health. According to Global Cancer Statistics 2020, the number of cervical cancer patients worldwide is approximately 604,127, including 56% of deaths, which shows an increasing trend over the next 12 years (1, 2). The prevalence of cervical cancer in China is also very serious (3, 4). In 2020, the number of new cervical cancer cases in China is 109,741 and the number of deaths is 59,060 (5). According to the latest data from the National Cancer Center, the incidence rate of cervical cancer is 11.4 per 100,000, and the mortality rate is 3.4 per 100,000, with an increasing trend for both (6). Due to the relatively high morbidity and mortality and large population base, the cervical cancer disease burden in China accounts for 18.2% of the global burden (1). In addition, the distribution of cervical cancer varies considerably between developed and developing countries and between rural and urban areas (7). The high incidence of morbidity and mortality and the health inequities caused by the disease have led to widespread concern about cervical cancer prevention and control. In fact, cervical cancer is currently a unique cancer with a clear cause that can be prevented and eliminated (7–9).

A “global strategy to accelerate the elimination of cervical cancer as a public health problem” was proposed at the WHO Assembly in 2020, and 194 countries reached consensus on the elimination of cervical cancer. The report emphasizes human papillomavirus (HPV) vaccination, cervical cancer screening, and precancerous lesion treatment to prevent and control cervical cancer (7). The three measures described above are cost-effectiveness measures and are listed in the health plans of Member States of the Global Action Plan for the Prevention and Control of Noncommunicable Diseases 2013-2020. For every $1 invested in cervical cancer prevention and control, the economy obtains a return of $3.20, a figure that rises to $26 when social benefits are taken into account. However, the achievement of cervical cancer management has not been satisfactory, especially due to difficulties in expanding measures to a large population.

As one of the countries committed to eliminating cervical cancer, China has made some progress in cervical cancer prevention and control, but we are a long way from the goal of eliminating cervical cancer. The purposes of this study were to review the research related to HPV vaccination, cervical cancer screening, precancerous lesion treatment and model prediction of cervical cancer elimination, to clarify the effects of different prevention and control measures and the influencing factors of service utilization, to analyze the challenges of cervical cancer elimination in China with the current prevention and control status, and to propose recommendations and strategies for the elimination of cervical cancer in China.

## 2 Preventive interventions of cervical cancer

Cervical cancer can be effectively prevented by HPV vaccination and screening. The effectiveness varies between HPV vaccine types and screening techniques. In addition to the vaccine and screening interventions themselves, utilization of both services also has an important impact on effectiveness. To further clarify strategies to eliminate cervical cancer, it is necessary to understand the characteristics of different screening methods and HPV vaccination. It is also important to specify the factors influencing service utilization for HPV vaccination and screening to understand why these cost effective measures are not working as well as they should.

### 2.1 HPV vaccination

There are three types of HPV vaccines, bivalent, quadrivalent and nine-valent, all of which have been proven to have good efficacy, longer-lasting protective effectiveness and benefits (10, 11). Bivalent HPV vaccine could significantly reduce the incidence of cervical intraepithelial neoplasia(CIN) grade 3 and cervical cancer (12). The relative reduction in the incidence of cervical cancer and CIN3 at age 12-13 was 87% and 97%, respectively. The incidence of invasive cancer can be greatly reduced by quadrivalent HPV vaccination, with vaccination before age 17 decreasing the incidence of invasive cancers to 0.12 (per 100,000 population). 1 dose of quadrivalent HPV vaccine in 10 years provides comparable protection efficacy against HPV16 and HPV18 with two and three doses of vaccination (13). The 9-valent vaccine has been shown to be equivalent to the quadrivalent in preventing the four types of HPV infections, as well as preventing five other types of HPV infections (14). In addition, HPV vaccination not only reduces the incidence of HPV16 and HPV18 infections, precancerous lesions and cervical cancer but also decreases the occurrence of genital warts and HPV-related tumors (15, 16).

HPV vaccination is influenced by a number of factors, and vaccination rates are limited on the global average. Only 55% of countries committing to eliminating cervical cancer have introduced HPV vaccination, and the HPV vaccination rate (calculated by world population)in 2019 was approximately 15% (17). HPV vaccination is impacted by trust in the vaccine, availability, price, etc (18). For example, the crisis of confidence in the HPV vaccine in Japan has led to very low vaccination rate (19). The HPV vaccination rate for adolescents in the United States in 2020 was 58.6%, with those below the poverty line having a higher rate than those above the poverty line (20). And this was associated with greater access to the childhood HPV vaccination program for those below the poverty line. Due to external funding support (21), Free HPV vaccination can be widely implemented in Rwanda with vaccination rates exceeding 90% (22).

### 2.2 Cervical cancer screening

Currently, the common screening methods are HPV testing, cytology screening and visual inspection with acetic acid and lugol iodine(VIA/VILI). Studies related to cervical cancer screening have focused the effectiveness of different screening techniques and triage strategies for HPV positive results (23, 24). Screening techniques that are often compared are HPV testing and cytology screening.

Cytological screening has been proven over decades to be effective in decreasing the incidence and mortality rate of cervical cancer (25–27). The advantages of this screening method are the relatively high specificity of the results due to diagnosis by professional pathologists and the relatively low price. Because of dependence on pathologists, cytology screening is not suitable for application and promotion in areas with insufficient health resources (28).

HPV testing has been shown to have good sensitivity and positive predictive value (29, 30), which is the first recommended method by WHO in the latest screening guidelines (31). Compared to cytology screening, HPV testing can provide 60-70% higher protective rate against invasive cervical cancer (32). An randomized controlled study showed that HPV testing was superior to cytological screening and VIA/VILI for detection of CIN2+ and CIN3+ (33). Another study in China with a population of approximately 1.16 million also confirmed that HPV testing was superior to cytology screening in detecting CIN2+ (34). Combined HPV testing and cytology screening provides better results than testing with either modality alone; however, the increased sensitivity compared to HPV testing alone is not obvious (35). HPV testing with HPV16/18 genotyping plus cytology triage can significantly improve CIN2+ detection rates by 34-36% in women aged 35-54 years compared to cytology screening (36).

In addition to screening technology, the willingness to utilize screening services also directly affects the effectiveness of cervical cancer prevention and control. Women’s health awareness, cognitive level, access to services, screening quality, follow-up management, and adequacy of funding for screening programs all influence the utilization of screening services (37). There are some differences in the factors influencing the utilization of cervical cancer screening services between developed and developing countries. In high-income countries, there are relatively few cases of non screening due to lack of access to screening, mainly due to lack of knowledge and health awareness (38). In contrast, women in most low- and middle-income countries (LMICs) have low levels of education, awareness, and access to screening. Utilization of screening services in many areas is affected by local sociocultural factors (39). These reasons contribute to regional disparities in cervical screening coverage, particularly low screening coverage in LMICs (40, 41).

## 3 Implementation of HPV vaccine and cervical cancer screening interventions: modeling predictions

Modeling predictions have become a standard method for developing public health measures worldwide, as well as for developing strategies to eliminate cervical cancer (42). Elimination of cervical cancer means reducing the incidence of cervical cancer to 4 per 100,000 or below, which is the most widely used of several thresholds (43). To provide evidence and recommendations for global cervical cancer prevention and control, the WHO created the Cervical Cancer Elimination Modeling Consortium (CCEMC), which uses modeling predictions to answer a range of questions about thresholds, strategies, timing, and the number of deaths that can be avoided by eliminating cervical cancer.

The two most representative studies are modeling predictions for 181 countries and 78 LMICs. Broad coverage of HPV vaccination and cervical screening has the potential to avert up to 12-13 million cervical cancer cases over the next 50 years, and a model study based on 181 countries found that the goal of cervical cancer elimination could be achieved in all countries by 2100 (44). The strategy is to implement HPV vaccination and two HPV screenings to cover 80%-100% of the population. The results of the 181-country modeling projection study were generally consistent with the timing and strategies for eliminating cervical cancer from the 78-country modeling projection study (43). By implementing the WHO’s 90-70-90 strategic goals, 99% of cervical cancer deaths could be avoided, and 62 million lives would be saved in the next century (45).

To select the most appropriate strategy, modeling predictions to eliminate cervical cancer have been carried out in different countries (**Table 1**). With HPV immunization and HPV screening, Australia has the potential to be the first country to virtually eliminate cervical cancer in 2028 (46), and Norway may reach its goal by 2039 (47). The United States is likely to reach the cervical cancer elimination threshold by 2038-2046 based on current cervical cancer screening coverage and HPV vaccination rates. The WHO goal of cervical cancer elimination could be achieved 10-13 years earlier if cervical cancer screening were expanded. Moreover, the study found that compared to the insignificant effect of increased HPV vaccine coverage on eliminating cervical cancer, the effect of increased screening coverage was more significant (48). Japan has a very low HPV vaccination rate due to the impact of the vaccine confidence crisis (19), and the goal of cervical cancer elimination is expected to be achieved by 2055-2060 if cervical cancer screening and HPV vaccine coverage are expanded (43, 45). Increasing HPV vaccine coverage is important to avoid cervical cancer deaths, yet it will not eliminate cervical cancer, and the goal of cervical cancer elimination in Japan can only be achieved by increasing cervical cancer screening coverage based on increased HPV vaccine coverage (49).

**Table 1:** Modeling studies for the elimination of cervical cancer.

Country	Author	Year	Model	Timeframe for the elimination of cervical cancer (<4 per 100,000)	Strategy for the elimination of cervical cancer
78 LMICs	Brisson	2020	HPV-ADVISE Harvard Policy1-Cervix	2059-2102	HPV vaccination (routine vaccination of girls aged 9-14 years, 90% coverage) + two HPV screenings (twice per lifetime at the ages of 35 and 45)
181 countries	Simms	2019	Policy1-Cervix	2055–2059 (very high HDI countries), 2065–2069 (high HDI countries), 2070–2079 (medium HDI countries), 2090–2100 onward (low HDI countries), 2070–2079 (worldwide mean)	HPV vaccination (80%-100% coverage) + two HPV screenings (twice per lifetime at the ages of 35 and 45)
Australia	Hall	2019	Policy1-Cervix	2028 (2021–2035)	Maintain the current HPV vaccination levels (coverage for those aged 12 years from 2012 onward was assumed to be 82.3% in females and 75.5% in males) + HPV screenings (every 5 years)
United States	Burger	2020	Harvard Policy1-Cervix	2038 (Harvard model), 2046 (Policy1-Cervix model), 10–13 years earlier (elimination year 2028 for the Harvard model vs. 2033 for Policy1-Cervix) if screening coverage is up to 90%	The status quo scenario HPV vaccination (approximately 65% by age 17 for females and 55% by age 17 for males, approximately 75% by age 26 for females and 62% by age 21 for males) + screenings (cytology screening every 3 years was recommended among women aged 21–65 years, 14% of women were assumed to never participate in screening)
Japan	Simms	2020	Policy1-Cervix	2055-2060	Simulation scenarios, screening and vaccination coverage were substantially increased by 2020. If the vaccine crisis continues, 9300–10800 preventable deaths will occur in the next 50 years (2020–69) due to cervical cancer.
China	Xia changfa	2019	Hybrid models (consisted of a deterministic age-structured compartmental dynamic model and an individual-based stochastic Monte Carlo simulation model)	2072 (2070–74) in urban China and 2074 (2072–76) in rural China (the current budget), 2063 (2059–66) in urban China and 2069 (2066–71) in rural China (double the current budget), 2057 (2053–60) in urban China and 2060 (2057–63) in rural China (no budgetary restrictions)	Strategy under the current budget, vaccination of 95% coverage for girls aged 12 years, and expanding coverage of once in a lifetime screening for women aged 45 years to 90% in urban areas and 33% in rural areas). With no budgetary restrictions, girls aged 12 years and coverage of screening at 5-year intervals for women aged 35–64 years was maximized.

**Table 1** Modeling studies for the elimination of cervical cancer

Predictive modeling analysis for the elimination of cervical cancer has also been performed in China. One study examined the time to elimination of cervical cancer in urban and rural areas under different budgets. Under the current budget, the best strategy to achieve the goal of eliminating cervical cancer is to have 95% HPV vaccine coverage for 12-year-old girls and 90% and 33% population coverage for once-in-a-lifetime screening for 45-year-old women in urban and rural areas, respectively (50). If the optimal strategy is implemented under the current budget, it is expected that the goal can be achieved in the early 2070s. In addition, 7.5 million cervical cancer cases and 2.5 million cervical cancer deaths could be avoided by 2021-2100, and 100 billion lives could be saved (51).

## 4 Problems and challenges of cervical cancer prevention and control in China

Several modeling studies have shown that cervical cancer can be eliminated through the combined implementation of HPV vaccine and screening (43, 44, 50), but the process of eliminating cervical cancer faces many challenges. Even in high-income countries, the 90-70-90 coverage target has not been achieved (52). For developing countries, the main challenge is the struggle between different interventions, coverage targets and costs (22). A systematic review covering 202 countries showed that 84% of women in high-income countries had been screened for cervical cancer, compared to 9% in LMICs (53). The cost of interventions has also affected China, where coverage of the HPV vaccine and cervical cancer screening is relatively low (54). To achieve the goal of elimination of cervical cancer in China, there is still a need to further clarify the problems and factors affecting service utilization in cervical cancer screening, HPV vaccination and precancerous lesion treatment.

### 4.1 Cervical cancer screening in China

Cervical cancer screening services have been transformed from nonexistent to available, but coverage remains limited. China’s cervical cancer prevention and control measures were conducted in 2009 and named the “two cancers” screening program, including breast cancer and cervical cancer. In addition, screening services have been integrated into basic public health services since 2019. Screening is free for all women aged 35-64 years, but due to factors such as insufficient funding and a large population to be screened, the state currently adopts the practice of providing a certain number of free screening services to rural women each year. Based on the official data from 2009 to 2020, the nationwide cumulative number of people screened for cervical cancer is approximately 130 million, while the number of people to be screened is much greater than that. The coverage of the current screening population is approximately 25.7%, and there is a huge gap in comparison to the requirement of over 70% coverage (50) (**Table 2**). One study showed lower coverage rates in rural areas than in urban areas (55).

**Table 2:** Current status of cervical cancer prevention in China and the WHO targets.

Intervention	Coverage	WHO Goal	Primary Public Health Services
HPV vaccination	<5%	≥90%	No
Cervical cancer screening	<30%	≥70%	Yes
Treatment of precancerous lesions	≈90%*	≥90%	No
Incidence	≥10/100000	<4/100000	——

Additional information: * The treatment rate of CIN2/3 was 95% and 89% in urban and rural areas, respectively.

**Table 2** Current status of cervical cancer prevention in China and the WHO targets

The affordability and accessibility of screening services are key factors impacting screening utilization by Chinese women (56), which further affects the coverage rate. Through a large-scale institutional survey, Li found that the capacity of LBC testing and pathology service provision in rural China is less than half of that in urban areas, with the most significant differences in pathology services (57). More importantly, according to the technology and efficiency of current screening, some may not be able to access one screening service for their lifetime. In addition, state financial assistance is far from sufficient to cover the cost of the program (58). Therefore, this will affect the quality of services provided by primary health facilities, which will also further affect women’s expectations and utilization of screening services. In terms of personal factors, income, health insurance, and health knowledge can also influence an individual’s utilization of services (59).

### 4.2 HPV vaccination in China

Domestic HPV vaccine production, development and vaccination are actively promoted, but coverage remains very low. There are three types of HPV vaccines: bivalent, quadrivalent and nine-valent, which became available in mainland China in 2016, 2017 and 2018, respectively, and all three vaccines are imported and self-funded (60). The bivalent domestic HPV vaccine became available in 2019 and is self-funded, and there are some high valent vaccines in clinical trials. HPV vaccination is not yet included in the National Immunization Program (NIP), which significantly affects the HPV vaccine coverage rate (61). The first city in China to carry out free HPV vaccination was Erdos, where the work started in August 2020 (62). Currently, more than a dozen cities are conducting HPV vaccination pilots. Most people need to make an appointment in advance and wait 3-6 months for vaccination services. It is often more difficult to make an appointment for the 9-valent HPV vaccine. The current HPV vaccination rate in girls aged 9-14 years in China is less than 5%, in contrast to the WHO vaccine coverage goal of over 90% (50) (**Table 2**).

The main factors influencing HPV vaccination are awareness of the vaccine, the price of the vaccine, and the availability of the vaccine (61, 63, 64). These are related to the late production and development of vaccines (65). There is a lack of awareness and knowledge about HPV vaccination among Chinese people (66, 67), which is related to factors such as education, income and occupation. The awareness of those employed in health work is relatively high (68), but there is a gap compared to developed countries (69, 70). The literature shows that the population has a relatively high propensity for HPV vaccination, but the price dampens the demand for the vaccine (71). The domestic bivalent vaccine is priced at 329 RMB for one dose, and the full vaccination costs approximately 1000 RMB. The group with the best vaccination effect is junior high school students (72, 73), whose parents accept the price of domestic and imported vaccines at 130 RMB and 166 RMB, respectively, which is far below the market price of vaccines. Parental acceptance of the HPV vaccine could increase to 61.3% if the vaccine is incorporated into the NIP (74). Moreover, the HPV vaccine is in short supply due to the long production cycle and slow approval process (75). This shortage of supply is most evident in the 9-valent HPV vaccine.

### 4.3 Treatment of cervical cancer and precancerous lesions in China

Regarding the treatment rate of precancerous lesions, there was no significant difference between rural and urban areas. The treatment rates of CIN2/3 were 95% and 89% in urban and rural areas, respectively (50). Overall, treatment rates for rural and urban CIN2/3 are close to or have reached the WHO target. However, there is a gap in cervical cancer treatment coverage in China compared to developed countries (76). High treatment rates are not necessarily due to positive cervical cancer screening results that force patients to seek further treatment. Many women have already missed out on cervical cancer screening (77), and they seek health care and treatment when they are not feeling well. One study showed that at least half of patients with CIN1 were lost to follow-up (50). The management of cervical cancer prevention and control in China has not formed a closed-loop management of screening, follow-up and treatment, which requires the joint efforts of the government and institutions.

## 5 Strategies to eliminate cervical cancer in China

### 5.1 Develop health policies at the national level to promote broad population coverage and sustainability of interventions: integrate interventions into basic public health services

Strategic policies must be developed to promote cervical cancer prevention and control in a comprehensive manner. Cervical cancer prevention and control receive unprecedented attention and support from the Chinese government. Health has been made a priority development goal and integrated into all policies. Increasing cervical cancer screening coverage has become a policy goal, which is clearly stated in the Outline for Women’s Development in China (2021-2030) as well as in the Healthy China initiative (2019-2030) (78, 79). In 2023, the National Health Commission of the People’s Republic of China released an action plan to accelerate the elimination of cervical cancer, specifying the goals to be achieved by 2025 and 2030 (80). In the next step, the policy should focus on the evaluation of the effectiveness of prevention and control, especially the evaluation of the effectiveness of the coverage of the three measures.

Prevention and control measures should be integrated into basic public health services. Although modeling predictive analysis provides strategies to eliminate cervical cancer, health policies are required at the national level to ensure that interventions are widespread and sustained (81). In the WHO Global Strategy for the Eradication of Cervical Cancer, primary health care is repeatedly mentioned as the best way to address public problems (82). This means that cervical cancer interventions should be widely provided at the primary health care level, which would improve the accessibility of services.

High coverage of cervical cancer interventions is achievable. This is not entirely determined by national economic capacity, despite the relatively high coverage of interventions in high-income countries. Data from 2018 show that cervical cancer screening coverage in the United States is 80.5%, while the vaccination rate for adolescents is less than 40% (83). Rwanda has implemented a national cervical cancer screening program and included the HPV vaccine in its immunization schedule, and its HPV vaccine coverage has exceeded 90% (84, 85). Therefore, even with limited costs, China has the potential to achieve the 90-70-90 strategic goals.

### 5.2 Improve cervical cancer screening coverage in China: large-scale application of AI-assisted cytology screening to rapidly increase screening coverage

As one of the countries committed to eradicating cervical cancer, cervical cancer screening is currently the focus of Chinese government interventions. Cytology screening is the main screening modality for cervical cancer in China, but it is difficult to meet the screening needs of a large-scale population. Cytology diagnosis relies on pathologists, and the lack of pathologists affects not only cytology screening services but also pathology biopsy services (57) Although HPV testing has the advantage of high sensitivity and relatively objective test results, further evidence is needed to support whether HPV testing can be performed on a large scale in China (86). To some extent, artificial intelligence-assisted cervical cancer cytology screening technology, which has emerged in recent years, avoids the disadvantages of the above two screening methods.

AI technology has a wide range of applications in early cancer screening, and it has shown strong potential in cytology screening (87, 88). It can greatly improve the efficiency of cervical cancer screening and reduce errors caused by subjective factors (89, 90). A study of 0.7 million people demonstrated an overall compliance rate of 94.7% between AI-assisted diagnosis and pathologists’ diagnosis (91), and the sensitivity of AI diagnosis was higher as the grade of cervical lesions increased (92). Other research has also proven the excellent diagnostic performance of AI technology (93, 94). More importantly, the speed of AI-assisted diagnostic cytology screening is geometrically increased compared to traditional cytology screening, which can help to rapidly enhance screening coverage in a shorter period of time.

AI screening can also improve the accessibility of screening services, especially in areas with poor economic levels. In other words, AI technology could greatly contribute to improving the equity of cervical cancer screening services. Technicians at the primary health service institutions simply take samples from the cervix, which are sent to a specialized laboratory. Then, the laboratory’s AI technology assists the specialist pathologist in reading the slides to make the final cytological diagnosis. AI technology can also be applied to colposcopic diagnosis, and it has shown good diagnostic performance and adaptability in less economically developed areas (95, 96). NIH has also indicated that AI can help improve cervical cancer screening, particularly in areas with insufficient health resources (97).

AI-assisted cytology screening not only has the good diagnostic performance described above but also has relatively low cost and adaptability to mass screening (98). AI-assisted cytology screening is not completely dependent on the pathologist and saves more labor costs than traditional cytology screening. In terms of the cost of consumables, AI-assisted cell screening is lower than HPV testing. In the National Free Cervical Cancer Screening Program, cervical cancer screening is priced at 49 RMB a case, which includes AI cytology screening, colposcopy, pathology biopsy, and follow-up visits.

Unlike HPV testing technology, AI-assisted cytology diagnostic technology has been widely used in China, especially in the national cervical cancer screening program. In addition to China, this new screening technology has been applied in Pakistan, and in the future, this technology will also benefit more countries along the Belt and Road. The province with the most extensive use of AI-assisted cytology screening in China is Hubei Province, which has the second highest incidence of cervical cancer among all provinces in China. Full coverage of cervical cancer screening in Hubei Province means approximately 12.67 million women, including all women aged 35-64 in urban and rural areas. For the first time, AI technology will make full cervical cancer screening coverage a reality in provinces with over 10 million women in a short period of time.

### 5.3 Increase HPV vaccination rates in China: conduct a free vaccination pilot of a domestic bivalent HPV vaccine and explore funding models to gradually promote its inclusion in the NIP

An HPV vaccination pilot should be promoted to advance HPV vaccination into the NIP. The biggest challenge to improving coverage is that the vaccine is not included in the NIP (61). The Chinese government also intends to gradually include the HPV vaccine in the NIP. The strategy to promote HPV vaccine inclusion in the NIP is to accumulate evidence and experience through a pilot approach to progressively expand HPV vaccination coverage to all target populations. Inclusion in the NIP includes two basic requirements: one is the availability of a cost-effective vaccine, and the other is that the cost of the vaccine is affordable to the government. For the first requirement, the domestic bivalent HPV vaccine has good protective efficacy (99), which creates more possibilities for HPV vaccination to be included in the NIP (100, 101). For the second requirement, free HPV vaccination should be encouraged for cities with good economic conditions. The government can give certain financial subsidies or explore multifunding models for less economically developed areas.

HPV vaccine inclusion in the NIP has boosted both vaccination and screening rates. Inclusion in the NIP means that the HPV vaccine could be made available to girls for free, which would directly reduce the impact of vaccine prices. Few crises of confidence or vaccine hesitation regarding HPV vaccination in China have been found in previous studies. Moreover, the inclusion of the vaccine in the NIP confirms the vaccine’s safety. Studies have also supported that HPV vaccination rates can be significantly increased if HPV vaccination is included in the NIP (64, 74). In addition, HPV vaccination helps increase women’s utilization of cervical cancer screening services (102), which can further reduce the probability of cervical cancer in this group of women.

The supply of vaccines should be increased to lay the foundation for higher vaccination rates. Furthermore, the approval process for HPV vaccination should be optimized to promote the supply of vaccines. The choice of experimental endpoint indicators affects the progress of approval and the supply of vaccines (103). The primary function of vaccines is to prevent disease rather than infection, so we often use disease as an endpoint indicator. Chinese regulators mainly use two indicators of persistent 12-month infection and CIN2+ lesions as endpoints. However, the WHO has used persistent 12-month infection and persistent 6-month infection as endpoints (104, 105). One study showed that there was little difference in HPV vaccine efficiency when HPV infection persisted for 12 months, when HPV infection persisted for 6 months, and when CIN2+ lesions were used as endpoints (106). Therefore, China should select appropriate endpoints based on WHO guidance and study results to increase the supply of vaccines, especially the availability of quadrivalent and nine-valent vaccines.

Health knowledge and awareness should be improved to promote the utilization of HPV vaccination services. First, knowledge dissemination efforts should be expanded. Knowledge of the HPV vaccine among adolescent girls and their parents can be promoted through campus and community outreach. It is also possible to follow examples of campus publicity and campus vaccination programs from developed countries. Second, the incorrect behavior of residents should be corrected. Many Chinese residents believe that the higher the valent of the vaccine, the more effective it is in preventing cervical cancer. Therefore, they choose to wait for the 9-valent vaccine and miss the optimal age for HPV vaccination. However, the WHO has emphasized in its HPV vaccination position paper that bivalent, quadrivalent and nine-valent vaccines have approximately equivalent effects in protecting against cervical cancer (107). The Chinese government needs to clearly promote the idea that girls should be vaccinated as soon as possible before they have sex.

## 6 Conclusion

The Global Strategy to Accelerate Cervical Cancer Elimination identifies HPV vaccination, screening, and treatment and management of precancerous lesions as key measures to eliminating cervical cancer. HPV vaccination offers good protection and longer-lasting efficacy, while cervical cancer screening is cost effective. However, the HPV vaccination rate and cervical cancer screening coverage in China are limited and remain far from the target set by the WHO. Artificial intelligence-assisted cytology screening is a low-cost and high-quality screening method, and large-scale application of this technology is the most promising approach to rapidly increase screening coverage. The domestic bivalent vaccine has proven to be cost-effective, and conducting free vaccination pilots and exploring funding models to gradually promote its inclusion in the NIP for HPV vaccination is a key action to increase vaccination coverage.

## Author contributions

LJ reviewed the literature, wrote the manuscript and was mainly responsible for the framework design of the manuscript. MC and LY provided advice on the final version of the paper. All authors contributed to the article and approved the submitted version.
